# Does gender disparity exist in neurosurgery training? Evidence from a nationwide survey from Pakistan

**DOI:** 10.1080/10872981.2024.2310385

**Published:** 2024-01-30

**Authors:** Muhammad Shakir, Hammad Atif Irshad, Ahmed Altaf, Shamila Ladak, Hafiza Fatima Aziz, Syed Ather Enam

**Affiliations:** a Section of Neurosurgery, Department of Surgery, Aga Khan University Hospital, Karachi, Pakistan; b Medical Student, Medical College, Aga Khan University, Karachi, Pakistan; c Neuro-oncology Fellow, Section of Neurosurgery, Department of Surgery, Aga Khan University Hospital, Karachi, Pakistan; d Section of Neurosurgery, Department of Surgery, Aga Khan University Hospital, Karachi, Pakistan

**Keywords:** Gender disparities, neurosurgical training, working hours, burnout, mentorship opportunities

## Abstract

Gender disparities are prevalent in the neurosurgical field, particularly for female trainees, despite the growing demand for neurosurgeons. The situation is bleaker in low-and middle-income countries, where gender disparities among neurosurgical trainees have not been evaluated. We aimed to gauge the gender differences in opportunities and perceptions of neurosurgery training in Pakistan by comparing responses between males and females. A nationwide web-based survey was conducted in Pakistan, covering 22 College of Physicians and Surgeons of Pakistan (CPSP) accredited neurosurgery training programs. Convenience sampling was used with a pilot-tested questionnaire. Data analysis was performed using SPSS version 26. A total of 120 trainees participated in our survey. The mean age of the participants was 30.4 ± 4.1 years, with 29.2% females and 70.8% males. Concerns about gender equity were more among females (34.3%) than males (27.1%). Poor work-life balance was reported by more females (34.3%) than males (30.6%). Burnout due to working hours was strongly agreed by more females (54.3%) than males (35.3%). More females (40%) acknowledged sufficient mentorship opportunities versus males (25%). Female respondents (65.7%) worked 50–100 hours per week, less than males (69.4%). Satisfaction with surgical exposure was lower among females (2.9%) compared to males (18.8%). More females reported access to teaching courses (82.9% vs. 77.6% males) and neurosurgical conferences (85.7% vs. 80.0% males), cadaver workshops (17.1% vs. 12.9% males), morbidity and mortality meetings (88.6% vs. 82.4% males), case-based sessions (82.9% vs. 75.3% males), and radiology discussions (82.9% vs. 74.1% males). Our study highlights substantial gender gaps in neurosurgical training, concerns over working hours, burnout, mentorship, work-life balance, and gender equity. These findings underscore the necessity for interventions to rectify these disparities and foster gender equity in neurosurgical training.

## Introduction

There has been an increasing trend in women entering medicine since the 1980s [1]. In 2005, the number of women entering medical school was nearly equal to that of men and in 2017, the number of women surpassed the number of men entering medical school for the first time [1–3]. Despite such progress, the proportion of women in surgical training and academic leadership positions remains staggeringly low, making up 10–20% of the surgical workforce [1,2,4]. From 2000 to 2013, the male-to-female ratio in surgery decreased from 5.49 to 2.95 indicating a relative rate reduction of − 46.18% [5]. While the gender gap has slightly narrowed, it is still far from achieving the same degree of progress as it did in nonsurgical specialties. The situation is much worse in low- and middle- income countries (LMICs), where financial instability and cultural barriers further hamper progression. According to data from College of Physician and Surgeons of Pakistan (CPSP), only 14% of the surgeons who have completed training in Pakistan since 1967 are women [6].

Gender disparity in the surgical workforce is particularly evident in neurosurgery, where women currently account for only 19% of all board-qualified neurosurgeons globally [7]. In a recent survey distributed to the women neurosurgeon community globally, 43.5% of the respondents identified as the only woman neurosurgeon in their practice or department [8]. Consistent with previously reported statistics across surgical specialties, deficits are most seen in LMIC settings, where the percentage of women neurosurgeons ranges from as low as 0% to as high as 10.53%, and an estimated 23,300 additional neurosurgeons are needed to address the current neurosurgical workforce deficit [9]

Studies across the globe have revealed the various barriers that impede women’s entry and career progression in neurosurgery, notably in academic settings, including a lack of role models and mentorship, frequent encounters with sexism and/or harassment, poor work-life balance and cultural expectations, wage gaps, underrepresentation as authors in neurosurgery journals and conferences, and the ‘glass ceiling’ effect [6–14]. Women only comprised 12% of the matched neurosurgery residents during 2000–2009, with a much higher attrition rate of 17% compared to a 5.3% male attrition rate [15]. This is an important finding, especially after the 2008 white paper on the future of neurosurgery clearly stating limited exposure and fear of gender equities as significant barriers to neurosurgery for females [16]. These findings have also trickled down to female medical students and their perceptions of neurosurgical training. In a group of 104 medical students from the US, a significant number of females were less likely to consider a neurosurgery residency, and 89% agreed that there was a ‘glass ceiling’ due to inequality and adversity that would affect their training in a male dominated field. There was a similar sentiment among Indian female medical students, 87.4% of whom believe that lacking prospects and inadequate mentorship are barriers to pursuing a neurosurgical residency [9,17]. In another study conducted in Pakistan, only 2 out of 163 medical students listed neurosurgery as a potential career option. Interestingly, both of these students were males [18].

Pakistan has cultural norms and health systems similar to those of other LMICs in the South Asian region, predisposing females to similar barriers and motivations. Although medical students have been surveyed for their interest in surgical subspecialities in the past, no previous study has assessed and evaluated factors contributing to gender disparities at the residency level in Pakistan. Through this study, we aim to assess and compare perceptions of male and female residents currently training in neurosurgery in Pakistan. This data can further provide valuable insights into how their training experiences may influence workforce dynamics in the field of neurosurgery, especially in LMICs.

## Methods

### Study design and setting

A cross-sectional study in the form of a survey was conducted from April 2nd to 20 April 2023, involving neurosurgery trainees in Pakistan. Ethical approval was granted by the Ethical Review Committee (ID: 2023–8483–24152) of Aga Khan University, Pakistan. The study targeted neurosurgery trainees, including fellows and instructors, across the nation.

### Sample size

A total of 22 neurosurgery training centers accredited by the College of Physicians and Surgeons of Pakistan (CPSP) were identified [19]. Using OpenEpi [20], the sample size was calculated, considering 177 registered neurosurgery trainees across these centers [19]. This calculation, based on a 95% confidence interval and 5% confidence limits, indicated a required sample size of 122. A 10% margin of error was also calculated, thereby requiring a minimum of 134 responses. Notably, our study achieved a response rate of 89.6% (120 participants) of our intended sample size.

### Selection criteria

#### Inclusion criteria

Participant is a neurosurgery trainee at a CPSP-accredited institute.

#### Exclusion criteria

Non-neurosurgery trainees and participants who did not consent to being a part of the study.

#### Data collection tool

The research team created a customized survey in collaboration with cross-sectional study experts and the Aga Khan University Department of Surgery’s neurosurgery research unit. This strategy was justified by the lack of a validated survey instrument appropriate for our intended audience. Rigid panel review was used to demonstrate content validity, and Cronbach’s alpha coefficient – which came out to be 0.78—was used to evaluate dependability. Ten neurosurgical trainees participated in a pilot study to gather feedback. On the basis of this feedback, the response alternatives were subsequently modified with the intention of improving clarity.

The final survey (Supplementary File 1) included the following sections: demographic characteristics, training program characteristics, research experience, perceptions and future plans.

#### Sampling and data collection strategy

A convenience-based sampling strategy was employed to find participants. An anonymous Google Form was used to conduct the survey, and it was preceded by a consent form that described the goals of the study. An ambassadorship program was created to recruit volunteers which would eventually help the research team disseminate the survey. Data collectors were recruited countrywide for this operation. Social media and in-person encounters were used to diversify the online channels used for data collecting. We also sent emails to neurosurgical training programs throughout Pakistan to participate in our research.

#### Statistical analysis

Statistical analyses were conducted using International Business Machines (IBM) Statistical Package for Social Sciences (SPSS) version 26. Descriptive statistics were used to report the demographic characteristics found through the study. Normally distributed continuous data was reported as mean ± standard deviation, whereas categorical data for responses to the survey were reported as frequencies and percentages (n; %). The responses were divided into male and female and consequently, categorical variables were compared with gender using Chi-squared tests. A p-value <0.05 was considered as significant for all analyses.

#### Ethical considerations

A thorough explanation of the study’s goals, methods, and participant rights was provided to each participant. They received a copy of the informed consent form and were free to ask any questions they might have had. The study was entirely voluntary, and participants were free to leave at any moment without facing any repercussions. Names of participants were not gathered, and identifying information was deleted from survey responses to guarantee anonymity and confidentiality. The lead investigator and other investigators were the only ones with access to the password-encrypted file containing the data.

## Results

### Demographic characteristics

A total of 120 trainees participated in our study, including neurosurgery residents from all years, fellows, and instructors, with a mean age of 30.43 ± 4.186 years. The cohort consisted of 29.3% females and 70.8% males across Pakistan: Islamabad (5.8%), Khyber Pakhtunkhwa (19.2%), Punjab (56.7%), and Sindh (18.3%). The majority were from the Government sector—85.7% females and 80% males – while 14.3% females and 20% males were from the private sector. Most respondents were post graduate year (PGY) 1 to 6 residents; fellows and instructors each accounted for under 5%. Monthly salaries ranged for 51.7% between 50,000 to 100,000 PKR, with 43.3% earning 100,000 to 150,000 PKR. The details are shown in Table 1.

**Table 1.** Respondent demographics, year of training, monthly salary, and household income.

Variable	Female n (%) 35 (29.3%)	Male n (%) 85 (70.8%)	Total n (%) 120 (100%)
Age (mean ± SD)	29.14 ± 2.46	30.96 ± 4.63	30.43 ± 4.19
**Province**			
Islamabad (Capital)	2 (5.7)	5 (5.9)	7 (5.8)
KPK	1 (2.9)	22 (25.9)	23 (19.2)
Punjab	23 (65.7)	45 (52.9)	68 (56.7)
Sindh	9 (25.7)	45 (52.9)	22 (18.3)
Balochistan	0 (0.0)	0 (0.0)	0 (0.0)
Gilgit-Baltistan	0 (0.0)	0 (0.0)	0 (0.0)
**Sectors**			
Government	30 (85.7)	68 (80)	98 (81.7)
Private	5 (14.3)	17 [20]	22 (18.3)
**Year of Training**			
Instructor	0 (0.0)	4 (4.7)	4 (3.3)
Fellow	1 (2.9)	1 (1.2)	2 (1.7)
PGY1	6 (17.1)	13 (15.3)	19 (15.8)
PGY2	11 (31.4)	13 (15.3)	24 (20.0)
PGY3	6 (17.1)	16 (18.8)	22 (18.3)
PGY4	7 [20]	12 (14.1)	19 (15.8)
PGY5	1 (2.9)	12 (14.1)	13 (10.8)
PGY6	2 (5.7)	7 (8.2)	9 (7.5)
Other*	1 (2.9)	7 (8.2)	8 (6.7)
**Monthly Salary (PKR)**			
100,000–150,000	15 (42.9)	37 (43.5)	52 (43.3)
150,000–200,000	0.0 (0.0)	3 (3.5)	3 (2.5)
50,000–100,000	19 (54.3)	43 (50.6)	62 (51.7)
<50,000	1 (2.9)	2 (2.4)	3 (2.5)
**Monthly Household Income (PKR)**			
100,000–150,000	15 (42.9)	35 (41.2)	50 (41.7)
200,000–300,000	6 (17.1)	23 (27.1)	29 (24.2)
300,000–400,000	6 (17.1)	10 (11.8)	16 (13.3)
400,000–500,000	1 (2.9)	1 (1.2)	2 (1.7)
<100,000	7 (20.0)	14 (16.5)	21 (17.5)
>500,000	0 (0.0)	1 (1.2)	1 (0.8)

Post Graduate Year (PGY); *not specified by the participant; Pakistani Currency (PKR).

### Training program characteristics

**Figure 1.**
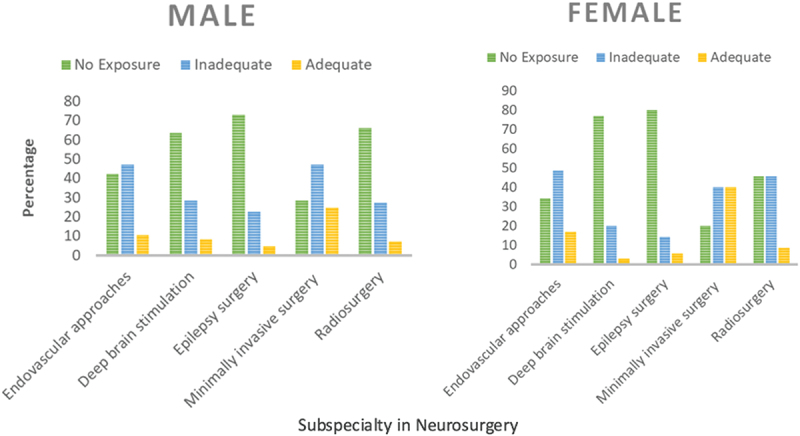
Comparison of exposure to subspecialty training between male and female neurosurgical residents.

Gender differences were observed in the responses to academic activities provided by training programs. Teaching courses: 79.2% (82.9% females, 77.6% males); cadaver workshops: 14.4% (17.1% females, 12.9% males); neurosurgical conferences: 81.7% (85.7% females, 80.0% males); live surgery workshops: 51.7% (54.3% females, 50.6% males); morbidity and mortality meetings: 84.2% (88.6% females, 82.4% males), case-based sessions: 77.5% (82.9% females, 75.3% males); radiology discussions: 76.7% (82.9% females, 74.1% males). Across all sessions, cranial, spinal, augmented reality simulations, and didactic lectures (22.5%, 23.3%, 9.2%, and 38.3%) were most commonly absent in training programs. Responses to educational activities offered by training programs are detailed in Table 2. Regarding exposure to neurosurgical subspecialties, the general trend leaned towards inadequate exposure to no exposure in most subspecialties. 82.9% of the females either had none or inadequate exposure to endovascular approaches compared to 89.5% males. Epilepsy surgery reported to have the lowest or worst exposure out of all the subspecialties with 94.3% females and 95.3% males reporting inadequate or no exposure. Comparatively, minimally invasive surgery was the subspecialty with the lowest no exposure rates, with 40% females and 24.7% males reporting adequate exposure. Radiosurgery had the second highest rates of no exposure with 45.7% females and 65.7% males indicating so, as shown in Figure 1. For hands-on surgical exposure (Table 3), similar percentages were noted amongst males and females, with the majority having completed less than 10 cases supervised 37.5% (37.1% females, 37.6% males) and unsupervised 21.7% (28.6% females, 18.8% males) per month.

**Table 2.** Neurosurgery training program characteristics.

	Female n(%)		Male n(%)		Total n(%)	
Does your training program offer?	NO	Yes	NO	Yes	NO	Yes
Teaching courses	6 (17.1)	29 (82.9)	19 (22.4)	66 (77.6)	25 (20.8)	95 (79.2)
Cadaver workshops	29 (82.9)	6 (17.1)	74 (87.1)	11 (12.9)	103 (85.8)	17 (14.2)
Neurosurgical conferences	5 (14.3)	30 (85.7)	17 (20.0)	68 (80.0)	22 (18.3)	98 (81.7)
Live surgery workshops	16 (45.7)	19 (54.3)	42 (49.4)	43 (50.6)	58 (48.3)	62 (51.7)
Cranial model-based simulation	27 (77.1)	8 (22.9)	66 (77.6)	19 (22.4)	93 (77.5)	27 (22.5)
Spinal model-based simulation	25 (71.4)	10 (28.6)	67 (78.8)	18 (21.2)	92 (76.7)	28 (23.3)
Augmented/Virtual reality simulation	31 (88.6)	4 (11.4)	78 (91.8)	7 (8.2)	109 (90.8)	11 (9.2)
**Educational Activities**						
Morbidity and Mortality Meetings	4 (11.4)	31 (88.6)	15 (17.6)	70 (82.4)	19 (15.8)	101 (84.2)
Tumor board meetings	11 (31.4)	24 (68.6)	32 (37.6)	53 (62.4)	43 (35.8)	77 (64.2)
Journal club	14 (40)	21 (60.0)	26 (30.6)	59 (69.4)	40 (33.3)	80 (66.7)
Case based sessions	6 (17.1)	29 (82.9)	21 (24.7)	64 (75.3)	27 (22.5)	93 (77.5)
Seminar	7 (20.0)	28 (80.0)	26 (30.6)	59 (69.4)	33 (27.5)	87 (72.5)
Preoperative discussion	11 (31.4)	24 (68.6)	18 (21.2)	67 (78.8)	29 (24.2)	91 (75.8)
Didactic lecture	26 (74.3)	9 (25.7)	48 (56.5)	37 (43.5)	74 (61.7)	46 (38.3)
Radiology discussion and interpretation	6 (17.1)	29 (82.9)	22 (25.9)	63 (74.1)	28 (23.3)	92 (76.7)

**Table 3.** Research experience and hands-on surgical exposure on the basis of gender.

	Female n (%)	Male n (%)	Total n (%)
**Neurosurgery Publications **			
**PubMed Indexed **			
<5	7 (20.0)	22 (25.9)	29 (24.2)
5–10	5 (14.3)	4 (4.7)	9 (7.5)
>10	1 (2.9)	0 (0.0)	1 (0.8)
None	22 (62.9)	59 (69.4)	81 (67.5)
**Non- PubMed Indexed Journal**			
<5	14 (40.0)	34 (40.0)	48 (40.0)
5–10	1 (2.9)	5 (5.9)	6 (5.0)
>10	0 (0.0)	1 (1.2)	1 (0.8)
None	20 (57.1)	45 (52.9)	65 (54.2)
**Hands-on Surgical Exposure per month**			
**Supervised**			
<10 cases	13(37.1)	32(37.6)	45(37.5)
10–20 cases	11(31.4)	25(29.4)	36 [21]
>20 cases	9(25.7)	21(24.7)	30 [22]
None	2(5.7)	7(8.2)	9(7.5)
**Unsupervised**			
<10 cases	10(28.6)	16(18.8)	26(21.7)
10–20 cases	16(45.7)	39(45.9)	55(45.8)
>20 cases	6(17.1)	11(12.9)	17(14.2)
None	3(8.6)	19(22.4)	22(18.3)

### Research experience (Table 3)

Most trainees lacked a PubMed indexed publication 67.5% (62.9% females, 69.4% males), and a great proportion also lacked a non-PubMed indexed journal publication 54.2% (57.1% females, 52.9% males). 24.2% (20.0% females, 25.9% males) had less than 5 PubMed indexed publications while 7.5% (14.3% females, 4.7% males) had 5 to 10 publications in a PubMed indexed journal.

### Perceptions

**Figure 2.**
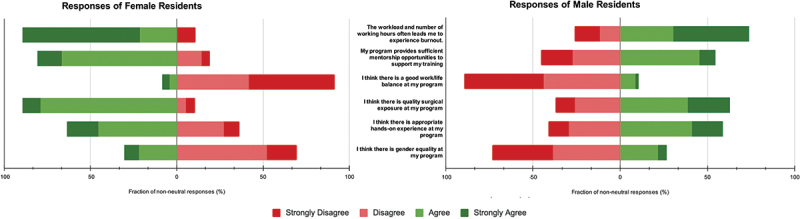
Depicts neurosurgery trainees’ perceptions, answered on a likert scale, to questions on working hours, mentorship, work life balance, surgical exposure, hands-on experience and gender equity.

All respondents were asked about work life balance, quality of surgical exposure, hands-on experience, and gender equity. When asked about the presence of a good work-life balance, 34.3% females and 30.6% males strongly disagreed, while for quality surgical exposure, a significant difference was seen between genders with 42.9% of females and 28.2% of males agreeing. For appropriate hands-on experience, 32.9% males, and 28.6% females agreed. When asked about working hours leading to burnout, 54.3% females and 35.3% males strongly agreed. Furthermore, 40% females and 29.4% males agreed on sufficient mentorship provided by their training programs. Gender equality at the workplace drew concern from 34.3% females and 27.1% males, though a minority fully endorsed gender equality (5.7% females, 3.5% males). Refer to Figure 2 for details.

### Future plans

When asked about which subspecialty the residents were planning to pursue, none of the female respondents chose epilepsy or peripheral nerve, while neurotrauma and critical care were found to be their top preferences (28.6%). Among males, there was at least a single response for each subspecialty and the most sought-after subspecialty was spine surgery (23.5%), followed by endovascular/interventional neuroradiology (14.1%). For country of choice for pursuing fellowship, most females, and males (45.7% and 43.5%) chose the United Kingdom (UK)/Ireland.

## Discussion

This is the first study in healthcare research in Pakistan to investigate the relationship between neurosurgical residents’ mentoring perceptions, scientific collaboration, and research performance in the context of gender. The findings of this study have highlighted significant gender disparities within the domain of progression indicators among residents in the field of neurosurgery. From a total of 120 neurosurgery residents, which make up 68% of the total residents of the field in Pakistan, merely 29.3% are females.

In a 2020 online survey encompassing all ACGME-accredited US neurosurgical programs, the findings revealed that females constituted 19.07% of neurosurgery residents across 115 programs, totaling 1510 residents [23]. Despite the higher proportion in comparison to the US, when the scarcity of neurosurgery residency programs in Pakistan is considered, an eminent gender gap in neurosurgical training has become the prevailing trend, both in developing and developed regions. The statistics become even more concerning when a comparison is drawn between the gender ratios at medical school and neurosurgery residency programs. Women in medical schools in the US constitute approximately 50% of the students [24], with a continuously rising trend [25]. In Pakistan, female students make up about 85% of the student body yet the persistence of gender gaps in specialty training remains evident [26].

Although the lack of publications amongst residents is a daunting statistic, the surprising gender equality in this matter is contrary to what has previously been observed. Although there has been a gradual increase in women’s representation in academic neurosurgical research, female authorship levels are still low even in developed countries, ranging from 13.3% to 19.9% [12]. Furthermore, only 3.6% of the research had females as senior authors and only 8.3% of female neurosurgeons have first-author abstract presentations [11,12]. This pattern indicates that even when females were granted research authorship, their involvement seldom extended to leadership roles. This gap also translates into poorer representation of women as editorial board members, contributing to 8.58% of all board members in neurosurgery journals and 5.53% in spinal journals [22,27]. This may be one of the reasons for decreased representation of women as article authors, in addition to the attrition of women in the advancement of neurosurgical careers. For example, female neurosurgeons held 15.4% and 13.3% of assistant and associate professor roles, but only 5.8% of full professorship roles [11]. Nevertheless, a significant increase in female first authorship was seen between 2003 and 2018, albeit not enough to bridge the current gap in authorship [28]. A 2021 Postgraduate Training Survey from Pakistan found poor research skills and inability to allocate time for research as main reasons for low participation amongst residents but did not compare the results between genders [29]. Future studies on our topic could focus further on the gendered aspect of trainee research such as barriers to publishing, funding restraints, and possible gender biases amongst senior authors.

Our findings also revealed more than half of the females (54.3%) compared to less than half of the male residents (35.3%) to agree with burnout due to their working hours. Past literature has shown a similar result with burnout being 60% more common in female health professionals [30] Women more often face the complex dilemma of balancing their professional aspirations with familial responsibilities, simultaneously fulfilling roles as both a mother and a spouse [21]. Fujimaki et al. reported that out of all female neurosurgeons in Japan, only 22% of women were able to take a maternity leave, 11% had to decrease from a full-time job, and some had to quit entirely [31]. While traditional gender roles are evolving, a gross outlook comparison with career still shows more responsibilities on females and thus could be a significant determinant in preventing females from achieving work-life balance. Our study further demonstrates that female respondents assessed the work-life balance as very poor and tended to disagree more with the existence of favorable work-life equilibrium in their training experiences. Similarly, most of the females from our study disagreed with gender equality being present in the workplace. Moreover, in the context of Pakistan, indigenous cultural norms have frequently imposed restrictions on women due to societal expectations, constraining their professional advancement in the workforce [32]. This is consistent with data from nearby regions in South Asia; a survey of female neurosurgeons from India showed that almost 73% of respondents were discouraged from entering neurosurgery likely because of the cultural expectations for women [10]. While previous literature has shown same gender mentor-mentee relations to be ideal, the underrepresentation of females in leadership positions may prevent female medical students from pursuing neurosurgery as a potential career [33]. In fact, women only constitute 8.85% of all neurosurgery residency program director (PD) positions [34].

Pahwa et al. analyzed neurosurgery subspecialty preferences, considering geographical and gender variables [17,35]. Their findings revealed a reduced prevalence of more specialized disciplines, such as stereotactic, functional, and peripheral nerve surgery, within developing nations. Regarding gender-related tendencies, the analysis indicated a proclivity among females towards pediatric neurosurgery [35]. Interestingly, in our study, neuro-trauma and critical care was the most popular choice among female residents.

As our study utilized a cross-sectional survey design, a more descriptive and subjective assessment of perceptions was not possible. A qualitative study is advised for a more in-depth assessment. Moreover, our sample consisted only of current trainees and therefore the relatively small sample size was a limitation, potentially leading to the absence of significant associations across various characteristics. Moreover, since residents’ decisions may evolve during their training due to various factors, our design cannot capture these changes comprehensively. Therefore, while we can estimate the contribution of specific factors to decision changes, we cannot confirm them definitively. As we did not delve into gender disparities at the provincial, institutional, or sectoral levels. These factors possess diverse resource availability and working conditions that could considerably influence gender disparities. Further research is necessary to comprehensively investigate this aspect.

### Recommendations

Addressing gender disparities in neurosurgery training in LMICs requires a multifaceted approach. It is crucial to establish mentorship programs by pairing female neurosurgery residents with experienced mentors to ensure guidance and support throughout training. It is also important to foster an atmosphere where everyone feels appreciated, neurosurgery departments should promote inclusiveness, tolerance, and open communication. Institutions should also adopt and enforce policies promoting gender equality and awareness campaigns should be launched to challenge gender stereotypes. In the social context for Pakistan, it is It is recommended to offer part-time training, job-sharing, or flexible hours to neurosurgical residents who are female and may have extra obligations at home. In addition, parental leave policies should be in place and communicated clearly to all trainees. By putting these suggestions into practice, we can start to close the gender gap in neurosurgical training in low- and middle-income countries and build a more diverse and equitable sector.

## Conclusions

The findings of our investigation demonstrate notable discrepancies and obstacles related to gender in the field of neurosurgical education. They have difficulties with work-life balance, burnout, mentoring, gender parity, and long hours. The results underscore the necessity of implementing interventions and establishing support networks to mitigate gender inequalities and foster parity in neurosurgical education. To develop targeted solutions and promote inclusive policies, more study is required into the multifactorial factors causing the reduced participation of women in neurosurgery residency training programs.

## Supplementary Material

Supplementary File.docx

## Data Availability

All analyzed data relevant to the study are included in the. Raw data that support the findings of this study are available from the corresponding author, upon reasonable request.
