# Supplementary material for: Does gender disparity exist in neurosurgery training? Evidence from a nationwide survey from Pakistan
Source: Med Educ Online. 2024 Jan 30;29(1):2310385. doi: 10.1080/10872981.2024.2310385 (PMC10829840; doi:10.1080/10872981.2024.2310385)
Supplement: Supplementary File.docx [file ZMEO_A_2310385_SM4344.docx]

**Supplementary File: Survey**

**Section 1: Demographic Characteristics**

1. Age?
2. Gender?
3. Male
4. Female
5. Other
6. Year of Neurosurgery Training
7. PGY1
8. PGY2
9. PGY3
10. PGY4
11. PGY5
12. PGY6
13. Fellow
14. Instructor
15. Other
16. In which province is your training institute/hospital?
17. Khyber Pakhtunkhwa
18. Punjab
19. Sindh
20. Balochistan
21. Gilgit Baltistan
22. Islamabad
23. Azad Jammu and Kashmir
24. Other
25. Which sector does your institute belong to?
26. Government
27. Private
28. What is your current monthly salary in Pakistani rupees?
29. Less than 50,000
30. 50,000-100,000
31. 100,000-150,000
32. 150,000-200,000
33. More than 200,000
34. What is your total monthly household income in Pakistani rupees?
35. Less than 100,000
36. 100,000-150,000
37. 150,000-200,000
38. 200,000-300,000
39. 300,000-400,000
40. 400,000-500,000
41. More than 500,000

**Section 2: Training Program Characteristics**

*Answer with Yes and No for Questions 1 and 2 in this section*

1. Does your training program provide:
2. Teaching courses
3. Cadaver workshops
4. Neurosurgical conferences
5. Live surgery workshops
6. Cranial model-based simulation
7. Spinal model-based simulation
8. Augmented/Virtual reality simulation.
9. Does your training program include:
10. Morbidity and mortality meetings
11. Tumor board meetings
12. Journal club
13. Case based sessions.
14. Seminars
15. Preoperative discussion
16. Didactic lecture
17. Radiology discussion and interpretation

**Section 3: Research Experience**

1. How many PubMed Indexed neurosurgery publications do you have in a peer reviewed journal?
2. None
3. Less than 5
4. 5-10
5. More than 10
6. How many non-PubMed Indexed neurosurgery publications do you have in a peer reviewed journal?
7. None
8. Less than 5
9. 5-10
10. More than 10
11. How many supervised hands-on surgical exposure have you had during your training per month?
12. None
13. Less than 10
14. 10-20
15. More than 20
16. How many unsupervised hands-on surgical exposure have you had during your training per month?
17. None
18. Less than 10
19. 10-20
20. More than 20
21. How has your exposure been with the following subspecialities in your training?

[Answer Q12 with one of the following options: Adequate, Inadequate, No Exposure]

1. Endovascular Approaches
2. Deep Brain Stimulation
3. Epilepsy Surgery
4. Minimally Invasive Surgery
5. Radiosurgery

**Section 4: Perceptions**

1. To what extent do you agree with the following statements:

[Answer Q1 with one of the following options: Strongly Disagree, Disagree, Neutral, Agree, Strongly Agree)

1. The workload and number of working hours often leads me to experience burnout.
2. My program provides sufficient mentorship opportunities to support my training.
3. I think there is a good work-life balance at my program.
4. I think there is good quality surgical exposure at my program.
5. I think there is appropriate hands-on experience at my program.
6. I think there is gender equality at my program.

**Section 5: Future Plans**

1. Does your institute offer fellowship training in neurosurgery?
2. Yes
3. No
4. In which subspeciality are you planning to pursue a fellowship?
5. Cerebrovascular
6. Endovascular/Interventional at Neuroradiology
7. Functional and Epilepsy
8. Neuro-trauma and Critical Care
9. Neuro-oncology
10. Pediatrics
11. Peripheral Nerve
12. Radiosurgery
13. Skull base/Complex Cranial
14. Spine
15. None
16. In which country are you planning to pursue a fellowship?
17. Australia
18. Germany
19. Pakistan
20. UK/Ireland
21. USA
22. None
